# Evaluation of mycotoxins in grains sold in Idah, Ajaka and Ogbogbo areas of Nigeria

**DOI:** 10.1099/acmi.0.000658.v3

**Published:** 2023-12-12

**Authors:** M.U. Ukwuru, A. Muritala

**Affiliations:** ^1^​ Department of Food Science and Technology, The Federal Polytechnic, Idah, Nigeria

**Keywords:** mycotoxin contamination, stored grains, recently harvested grains, food safety, control measures, risk assessment

## Abstract

Mycotoxin contamination in grains is a significant concern due to its adverse effects on human and animal health. Understanding the levels and patterns of mycotoxin contamination in different regions and storage conditions is crucial for developing effective control strategies. This study aimed to assess mycotoxin levels in stored and recently harvested grains in three regions (Idah, Ajaka, and Ogbogbo) and investigate the implications for food safety. The study involved the analysis of mycotoxin levels in maize, rice, sorghum, and millet using appropriate mycotoxin extraction method based on the mycotoxins of interest and a suitable HPLC system. The findings revealed the presence of mycotoxins such as aflatoxins (1±0.2–5±0.4 µg kg^−1^), deoxynivalenol (520±0.8–700±1.2 µg kg^−1^), zearalenone (200±0.4–370±0.6 µg kg^−1^), ochratoxins (2±0.2–4±0.3 µg kg^−1^), and fumonisin (0±0.0–4±0.3 µg kg^−1^) in both recently harvested and stored grains. Patulin was absent in most of the samples. Variations in mycotoxin levels were observed among different grains and regions, highlighting the need for targeted interventions. The European Commission mycotoxin standards in grains for human consumption are: aflatoxins 4 µg kg^−1^ in maize, millet and sorghum while rice is 8 µg kg^−1^, deoxynivalenal 1750 µg kg^−1^, zearalenone 100 µg kg^−1^, ochratoxin A 5 µg kg^−1^ for maize, rice and millet, then 10 µg kg^−1^ for sorghum. Fumonisin is 4000 µg kg^−1^ but no level for rice and patulin is 50 µg kg^−1^ for rice and none for the other grains. This study demonstrates the persistence of mycotoxin contamination in stored grains and the importance of considering specific crop types and geographical locations when addressing mycotoxin contamination. The findings underscore the significance of implementing effective control measures to mitigate mycotoxin contamination and enhance food safety. The study provides valuable insights into mycotoxin contamination and emphasizes the need for comprehensive risk assessment studies and appropriate regulatory measures.

## Data Summary

No new data, tools, software and codes have been generated and are not required for this work to be reproduced.

**Impact Statement**

This comprehensive study on mycotoxin contamination in grains from multiple regions provides valuable insights into the prevalence and levels of mycotoxins in both recently harvested and stored grains. The findings reveal significant variations in mycotoxin contamination across different grain types and regions, emphasizing the need for effective control measures to ensure food safety.

By identifying the mycotoxin levels in different grains and their variations during harvest and storage, this research provides important information for policymakers, food regulatory agencies, and farmers to develop targeted interventions and strategies for reducing mycotoxin contamination and mitigating associated risks.

Furthermore, the study highlights the potential health hazards associated with mycotoxin exposure through contaminated grains. The presence of mycotoxins, such as aflatoxins, deoxynivalenol, zearalenone, and ochratoxin A, in the analysed grains raises concerns about their adverse effects on human health, including carcinogenic, immunotoxic, and hepatotoxic effects. Understanding the extent of mycotoxin contamination in different regions enables stakeholders to implement preventive measures, such as good agricultural practices, proper storage facilities, and mycotoxin monitoring programmes, to safeguard public health.

The findings of this study also serve as a baseline for future research and monitoring efforts. By identifying knowledge gaps and areas of concern, this research provides a foundation for further investigations into the sources, factors influencing mycotoxin contamination, and the effectiveness of control measures. It calls for continued research and collaboration among scientists, agricultural practitioners, and policymakers to develop sustainable solutions that ensure safe and high-quality grain products.

This study’s impact lies in its contribution to the understanding of mycotoxin contamination in grains, its implications for food safety and human health, and the identification of areas requiring further attention. By raising awareness about mycotoxin contamination, the study serves as a catalyst for implementing preventive measures, promoting food security, and protecting public health.

## Introduction

Mycotoxins are toxic secondary metabolites produced by fungi that can contaminate various agricultural crops, including grains. These naturally occurring toxins pose significant concerns for both grain quality and human health. Mycotoxin contamination in grains is a global issue, impacting food safety, trade, and public health. Mycotoxins can have detrimental effects on grain quality, leading to reduced nutritional value, compromised organoleptic properties, and economic losses [1]. Furthermore, mycotoxin-contaminated grains can have severe consequences on human health when consumed either directly or through animal products derived from contaminated grains.

The consumption of mycotoxin-contaminated grains has been associated with a wide range of adverse health effects, including acute toxicity, chronic diseases, immunosuppression, and carcinogenesis [2, 3]. Some common mycotoxins of concern in grains include aflatoxins, ochratoxins, fumonisins, deoxynivalenol (DON), and zearalenone. Tarazona *et al*. [4] has highlighted the potential health risk associated with mycotoxins in oat grains consumed in Spain.

Recent research has highlighted the need for continued evaluation of mycotoxins in grains. For example, a study conducted in Europe found that mycotoxin contamination in cereal grains varied depending on the geographical region and crop type, emphasizing the importance of local assessments [5]. Similarly, a study in Nigeria reported significant levels of mycotoxins in locally consumed maize, highlighting the need for monitoring and mitigation strategies [6].

Understanding the occurrence and levels of mycotoxins in grains sold in specific regions, such as Idah and its environs – a grain producing hub in Nigeria, is crucial for assessing food safety risks and implementing appropriate control measures.

Mycotoxin contamination in grains is a significant concern in Idah, Nigeria, due to its potential impact on food safety and public health. Mycotoxins are toxic secondary metabolites produced by fungi that can contaminate grains during pre-harvest, harvest, and post-harvest stages [7]. However, there is a lack of comprehensive evaluation of mycotoxin levels in grains sold in this region, leading to uncertainty regarding the extent of contamination and potential health risks for consumers.

Numerous studies have highlighted the presence of mycotoxins in grains in Nigeria, including maize, which is a staple food in the country. Adetunji *et al*. [6] conducted a meta-analysis of survey reports and found significant mycotoxin contamination in various food commodities. Ezekiel *et al*. [7] investigated fungal and bacterial metabolites in stored maize from different agro-ecological zones in Nigeria, demonstrating the presence of mycotoxins in grains. These findings indicate the potential risk associated with mycotoxin exposure through grain consumption in Idah and its surrounding areas.

The consequences of mycotoxin contamination in grains can be severe. Mycotoxins pose significant health risks, including acute and chronic effects. For instance, deoxynivalenol (DON), a common mycotoxin, has been associated with immunosuppression and adverse effects on the liver [2]. Aflatoxins, another group of mycotoxins, are potent carcinogens and can increase the risk of liver cancer [3]. In addition to the potential health implications, mycotoxin contamination can result in economic losses for farmers and traders. Non-compliance with mycotoxin regulatory limits can hinder domestic and international trade, limiting market opportunities and revenue for grain producers [8]. Thus, it is crucial to evaluate grains in Idah for mycotoxin contamination to ensure food safety, protect public health, and facilitate trade in the agricultural sector.

To address this issue effectively, it is necessary to conduct systematic assessments of mycotoxin levels in grains sold in Idah, considering different grain types and storage conditions. By implementing regular monitoring programmes, appropriate mitigation strategies can be developed to minimize mycotoxin contamination and its associated risks for both consumers and the agricultural industry. Many novel technologies of food processing have been explored for mycotoxin decontamination [9]. Lu *et al*. [10] designed a sensitive and quantitative on-site detecting solution for Aflatoxin B1 (AFB1), Ochratoxin A (OTA) and Zearalenone (ZEN) as often found in mouldy grains. This proposed method is reported to sensitively detect AFB1, OTA and ZEN in low detection limits.

Food safety and public health are of paramount importance. Mycotoxin contamination in grains can have severe health effects, including liver damage, immunosuppression, and increased cancer risk [2, 3]. Evaluating mycotoxin levels in grains will provide crucial insights into contamination levels and potential health risks for consumers.

There is a knowledge gap specific to the Idah region. While studies have highlighted mycotoxin contamination in grains in Nigeria, there is a lack of comprehensive research focusing on the Idah region [6, 7]. This study will fill this gap by providing region-specific data, enabling better risk assessment and management strategies.

Mycotoxin contamination in grains has economic implications. Non-compliance with mycotoxin regulations can hinder domestic and international trade, leading to economic losses for farmers and traders [8]. Evaluating mycotoxin levels in grains sold in Idah will help identify contamination hotspots, inform mitigation strategies, and minimize economic losses associated with rejected or low-quality grain lots. Effective mitigation and intervention strategies are needed. Evaluating mycotoxins in grains will provide essential data for developing targeted control measures. Identifying sources and types of mycotoxin contamination will enable interventions at different stages of grain production, ensuring the production and availability of safe and high-quality grains.

Regulatory compliance and trade facilitation are important considerations. Compliance with mycotoxin regulations is crucial for domestic and international grain trade [8]. By evaluating mycotoxin levels in grains sold in Idah, this study will contribute to regulatory compliance and enhance trade relationships, ensuring the marketability of grains produced in the region. By evaluating mycotoxin contamination in grains sold in Idah, Nigeria, this study will provide valuable insights and data that can inform risk assessment, mitigation strategies, and regulatory compliance.

In this study, we employed the use of the HPLC methods, which are well-established and validated protocols for mycotoxin analysis and for its high sensitivity, allowing for the detection of mycotoxins even at very low concentrations. HPLC is highly selective, which means it can distinguish between different mycotoxins present in a sample as grains can be contaminated with various mycotoxins simultaneously. It allows for the quantification of mycotoxin levels in grains and can be used to detect and quantify a wide range of mycotoxins, including aflatoxins, ochratoxins, fumonisins, zearalenone, and many others. This versatility is valuable in comprehensive mycotoxin analysis. These methods for mycotoxin analysis are highly reproducible and are often recognized and approved by regulatory agencies, such as the FDA and the European Food Safety Authority. HPLC systems can handle multiple samples in a relatively short time, making them suitable for large-scale analyses that are often required in food safety and regulatory testing. Despite HPLC being a standard for mycotoxin analysis due to its comprehensive capabilities, fluorescence and optical spectroscopy can be valuable as rapid screening tools, especially when dealing with naturally fluorescent mycotoxins or specific mycotoxins with unique optical properties.

The purpose of this study was to evaluate mycotoxin contamination in grains sold in Idah, Nigeria, and its surrounding areas. The aim was to assess the levels of mycotoxins in different grain types and storage conditions, and to determine the extent of contamination, potential health risks, and economic implications. This study aims to provide region-specific data on mycotoxin contamination to inform risk assessment, develop mitigation strategies, ensure food safety, protect public health, and facilitate trade in the agricultural sector.

## Methods

### Study location

**Fig. 1.**
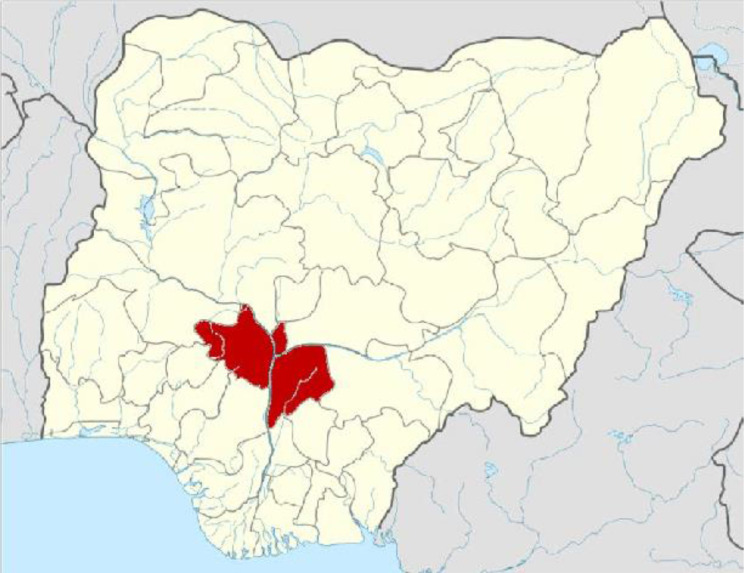
Map of Nigeria showing Kogi State in red [40].

**Fig. 2.**
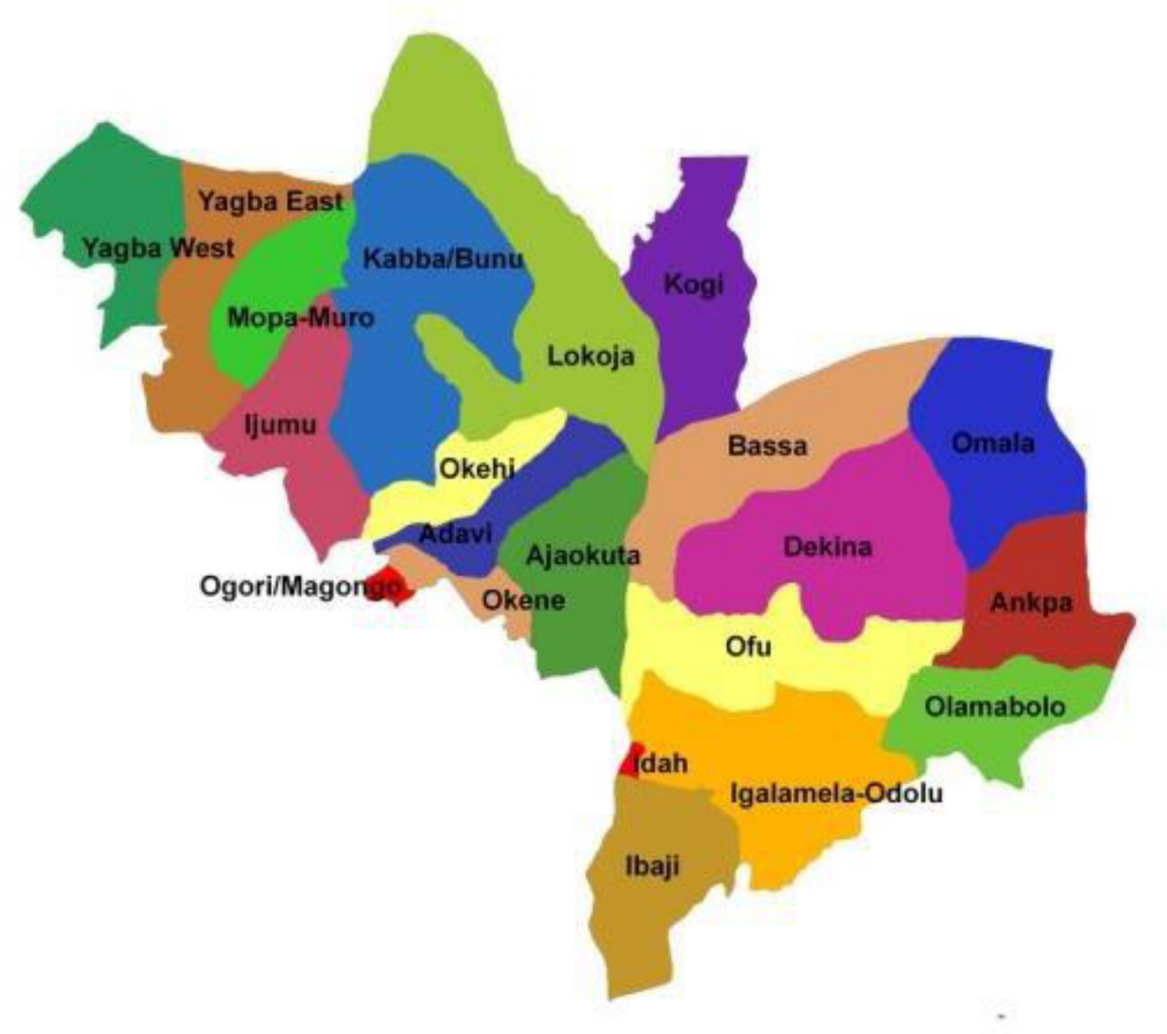
Map of Kogi State showing Idah and Igalamela-Odolu Local Government Areas [41].

This study was carried out in parts of Kogi State, Nigeria (Fig. 1). Idah is a town in Kogi State, Nigeria (Fig. 2), on the eastern bank of the Niger River in the middle belt region of Nigeria. Latitude: 7° 04' 60.00’ N Longitude: 6° 44' 59.99’ E, while Ajaka and Ogbogbo are in Igalamela-Odolu Local Government Area in Kogi State, Nigeria (Fig. 2). It is bordered by the Niger River in the west and Enugu State in the east. Latitude: 7° 10' 9.60’ N Longitude: 6° 49' 20.99’ E as shown on the maps. See the link http://www.maplandia.com/nigeria/kogi/idah/idah/ to view a satellite image of Idah, Ogbogbo and Ajaka.

### Study design

The study design involved a cross-sectional sampling approach to assess mycotoxin contamination in maize, sorghum, millet, and rice sold in Idah, Ajaka, and Ogbogbo in Kogi State, Nigeria. The study was conducted over a period of 2 years (2020–2021), considering seasonal variations in grain availability and potential fluctuations in mycotoxin contamination levels.

### Sample collection strategy

Markets and retail points in Idah, Ajaka, and Ogbogbo were randomly selected. Different areas within each location were fairly represented to capture the variability in grain sources. Thirty samples were collected from each location, considering factors such as grain consumption patterns, market volume, and desired level of confidence. Sample collection was carried out every 3 months throughout the study duration to capture potential temporal variations in mycotoxin contamination. Composite samples of each grain type were collected from various vendors or storage facilities within the selected markets immediately after harvest and during storage. Vendors were randomly selected to obtain representative samples. Upon collection using sterile low-density polyethylene, samples were labelled and properly stored in appropriate containers to prevent cross-contamination. Foreign materials or visibly contaminated grains were removed from the samples. Each composite sample was homogenized by thoroughly mixing the collected subsamples. This step ensured a representative subsample for subsequent analysis. The homogenized samples were divided into aliquots for different analyses to cover all desired mycotoxins and techniques. The subdivided samples were stored at 4 °C in a refrigerator to maintain sample integrity until analysis [11].

### Mycotoxin analysis using HPLC

The representative subsamples of grains obtained from the composite samples were weighed and ground. The sample was homogenized by thoroughly mixing the ground grain. A portion (5 g) of the homogenized sample was taken for further extraction and analysis. An appropriate extraction method based on the mycotoxin(s) of interest was chosen. Solvent extraction using organic solvent, methanol, for aflatoxin B1, B2, G1, G2. Solvent extraction using mixtures of methanol and water trichothecenes, e.g. deoxynivalenol, T-2 toxin, HT-2 toxin, zearalenone and its metabolites, ochratoxins, e.g. ochratoxin A and fumonisins. Liquid-Liquid Extraction (LLE) using organic solvent dichloromethane was employed for patulin extraction. All the extractions were performed in duplicate for each sample ensuring proper solvent-to-sample ratio, extraction time, and temperature. Extractions were carried out according to the established protocol of the European Commission [11]. Clean-up of the extracts was done using solid-phase extraction (SPE) following established methods [11] to achieve optimal removal of interferences. A suitable HPLC system (Thermo Scientific UltiMate 3000 HPLC System) equipped with Thermo Scientific Hypersil GOLD C18 Column, detectors, and software (Thermo Scientific Chromeleon Chromatography Data System) was selected [12]. The mobile phases were prepared, and the gradient programme was set up. The extracted and cleaned samples were injected into the HPLC system. The chromatographic conditions were optimized, including flow rate, column temperature, injection volume, and detection wavelengths, based on the mycotoxin(s) being analysed. Standards of known mycotoxin concentrations were run to generate calibration curves for quantification. Samples were analysed in triplicate to ensure precision and accuracy. Mycotoxins were identified and quantified by comparing retention times and peak areas with the corresponding standards. Thermo Scientific Chromeleon Chromatography Data System was used to calculate mycotoxin concentrations in the samples. Blank samples, spiked samples, and reference materials were used to validate the accuracy and precision of the analysis. System suitability tests were performed regularly to ensure the proper functioning of the HPLC system. Laboratory quality control protocols were adhered to including instrument calibration, method validation, and data verification. SPSS 23.0 (2015) software version (SPSS Inc., Chicago, IL, USA) was used to analyse the data. Data obtained are presented as mean±SD and subjected to analysis of variance (ANOVA). Means that differed significantly were separated by Tukey’s HSD test [13].

## Results and discussion

### Results

**Fig. 3.**
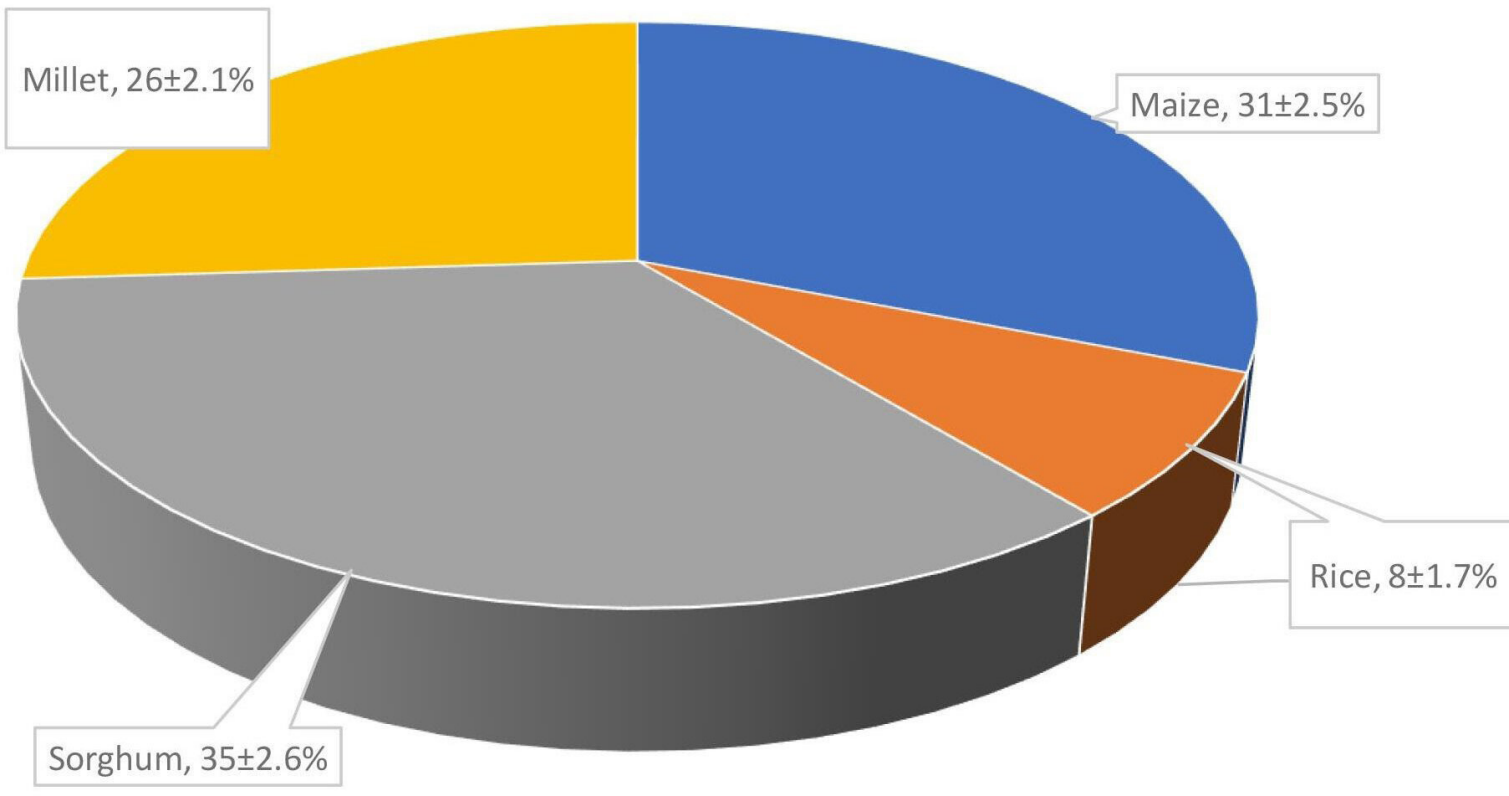
Percentage mycotoxin contamination of grains in Idah and adjoining region from 2020-2021.

The mycotoxin contamination levels in grains sold in the Idah region from 2020 to 2021 were assessed, and the results are presented in Fig. 3. The data indicate varying levels of mycotoxin contamination across different grain types.

Among the grains analyzed, sorghum exhibited the highest percentage of mycotoxin contamination at 35 %. This finding suggests that sorghum in the Idah region had a relatively higher risk of mycotoxin presence compared to other grains. Following sorghum, maize showed a significant level of mycotoxin contamination, with 31 % of samples testing positive for mycotoxins. Millet also demonstrated a considerable level of mycotoxin contamination, with 26 % of samples affected. These results highlight the need for careful monitoring and quality control measures to mitigate mycotoxin risks associated with millet in the Idah region. Rice exhibited the lowest mycotoxin contamination level among the grains assessed, with 8 % of samples showing mycotoxin presence. However, it is important to note that even low levels of mycotoxin contamination in rice can still pose health risks and requires ongoing monitoring.

**Fig. 4.**
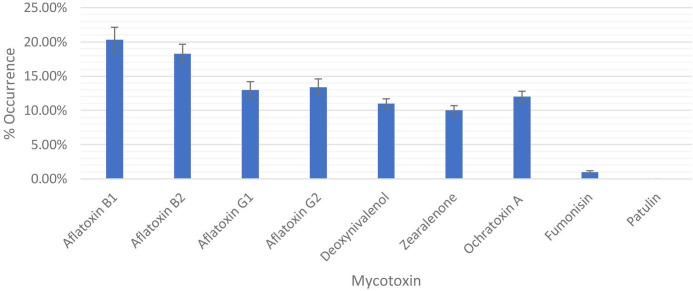
Percentage occurrence of mycotoxins on grains in Idah and adjoining region.

The occurrence of mycotoxins in grains in the Idah region was assessed, and the results are presented in Fig. 4. The data indicate the presence of multiple mycotoxins, with varying percentages of occurrence for each toxin.

Among the analysed mycotoxins, aflatoxin B1 had the highest occurrence at 20.34 %. Aflatoxin B2 and aflatoxin G2 were also prevalent, with occurrence percentages of 18.26 and 13.4 %, respectively. Aflatoxin G1 exhibited a slightly lower occurrence rate of 13 %.

Deoxynivalenol (DON), a mycotoxin commonly associated with *Fusarium* species, was detected in 11 % of the analysed samples. Ochratoxin A, known for its occurrence in various commodities, was found in 12 % of the grains. Zearalenone, a mycotoxin produced by *Fusarium* fungi, was present in 10 % of the samples. In contrast, fumonisin, another mycotoxin produced by *Fusarium* species, exhibited a relatively lower occurrence rate of 1 % in the analysed grains. Notably, patulin, a mycotoxin associated with certain moulds, was not detected in any of the samples. The results indicate a significant presence of aflatoxins, particularly aflatoxin B1 and aflatoxin B2, in the grains from the Idah region.

**Fig. 5.**
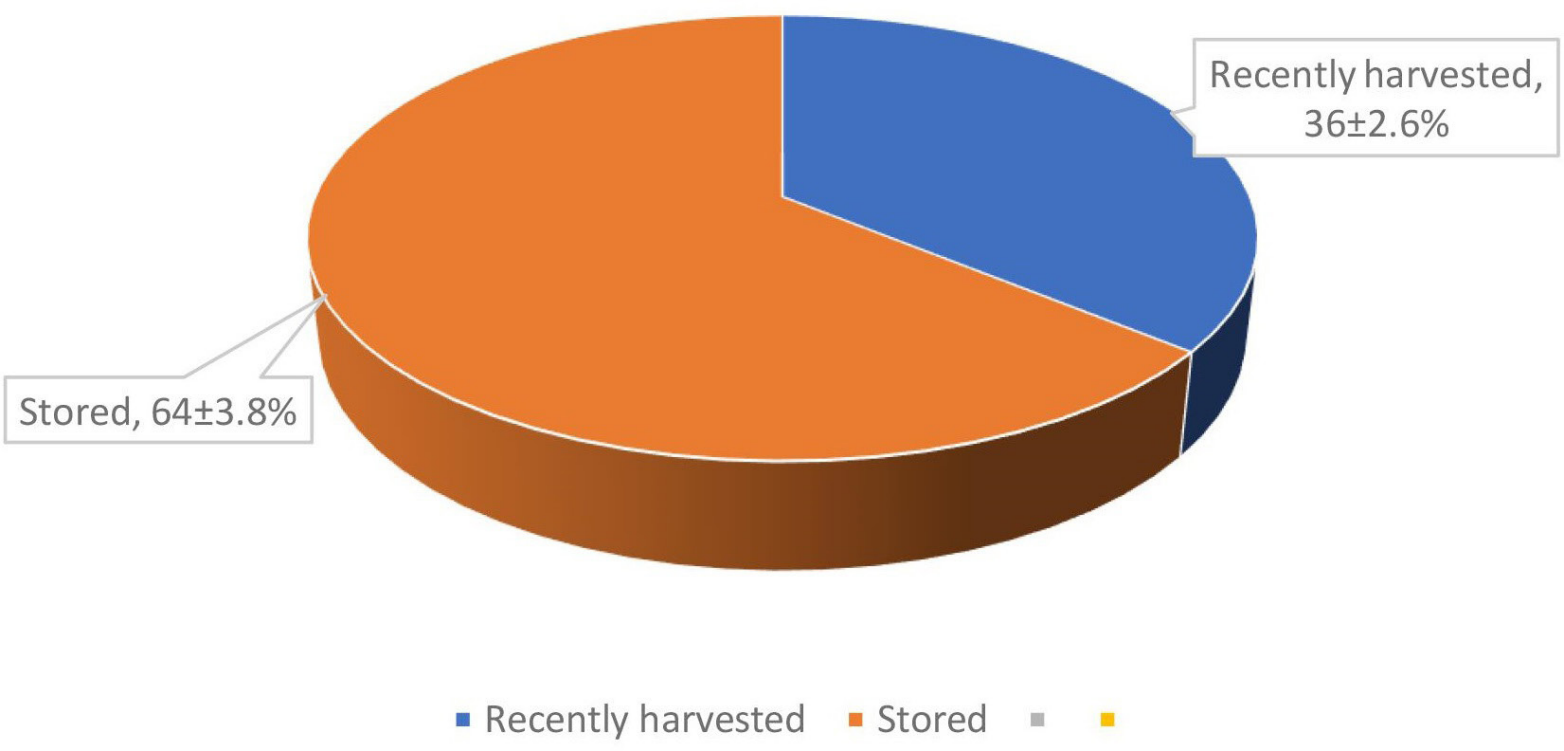
Percentage mycotoxin contamination of recently harvested and stored grains in Idah and adjoining region.

The data presented in Fig. 5 shows the percentage of mycotoxin contamination in grains during harvest and storage in the Idah region. It indicates that a significant proportion of mycotoxin contamination occurs during the storage phase (64%), while a relatively lower percentage is observed during the harvest (36%).

**Fig. 6.**
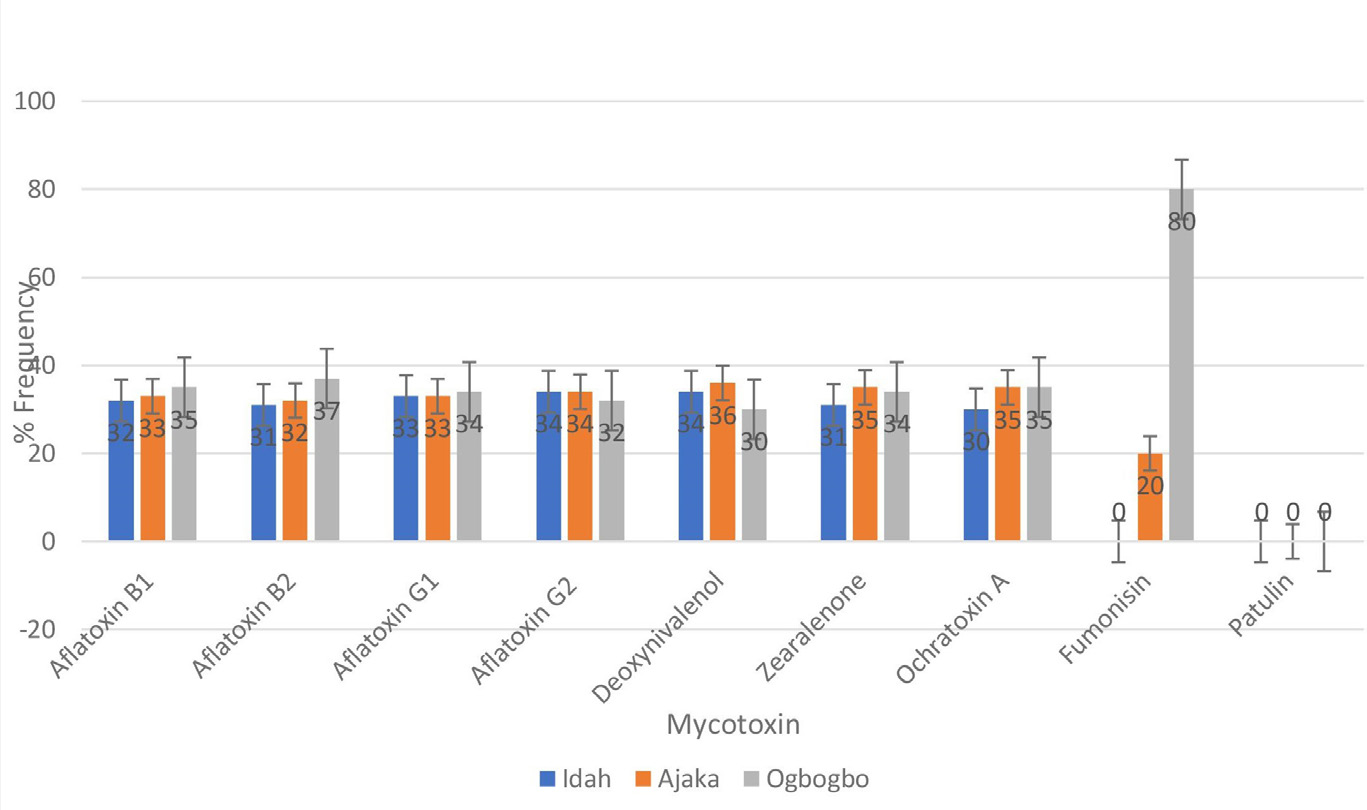
Location specific percentage frequency of mycotoxin contamination of grains in Idah and adjoining region.

The data presented in Fig. 6 shows the location-specific percentage frequency of mycotoxin contamination in grains in the Idah region. The results are based on a sample size of 250 and provide insights into the distribution of mycotoxins across three locations: Idah, Ajaka, and Ogbogbo.

Among the analysed mycotoxins, aflatoxin B1 exhibited a frequency of 32 % in Idah, 33 % in Ajaka, and 35 % in Ogbogbo. Aflatoxin B2 showed a similar pattern, with frequencies of 31, 32, and 37 % in Idah, Ajaka, and Ogbogbo, respectively. Aflatoxin G1 and aflatoxin G2 also displayed comparable frequencies across the three locations. Deoxynivalenol (DON), a mycotoxin associated with *Fusarium* species, exhibited frequencies of 34 % in Idah, 36 % in Ajaka, and 30 % in Ogbogbo. Zearalenone, another mycotoxin produced by *Fusarium* fungi, showed frequencies of 31 % in Idah, 35 % in Ajaka, and 34 % in Ogbogbo. Ochratoxin A, known for its occurrence in various commodities, exhibited frequencies of 30, 35, and 35 % in Idah, Ajaka, and Ogbogbo, respectively.

Interestingly, fumonisin, a mycotoxin produced by *Fusarium* species, was not detected in any of the samples from Idah but showed frequencies of 20 % in Ajaka and 80 % in Ogbogbo. Patulin, another mycotoxin associated with certain moulds, was not detected in any of the three locations. These results indicate variations in mycotoxin contamination across different locations within the Idah region.

Among the aflatoxins, aflatoxin B1 was detected in maize at a level of 1±0.2 µg kg^−1^, while aflatoxin B2 was not detected in any of the grains. Aflatoxin G1 was not detected in any of the grains, and aflatoxin G2 was detected only in millet at a level of 1±0.2 µg kg^−1^. Regarding trichothecenes, deoxynivalenol was found in maize at a level of 520±0.8 µg kg^−1^, while it was not detected in rice, sorghum, or millet. Zearalenone was detected in maize and sorghum at levels of 250±0.4 µg kg^−1^. Among the ochratoxins, ochratoxin A was detected in maize at a level of 3±0.3 µg kg^−1^, while it was not detected in rice, sorghum, or millet. Fumonisin and patulin were not detected in any of the grains.

The data in Table 1 indicate variations in mycotoxin levels among different grain types. Maize showed higher levels of mycotoxins compared to other grains, particularly in terms of deoxynivalenol and aflatoxin B1. These findings are consistent with previous studies that have reported higher mycotoxin contamination in maize due to its susceptibility to fungal infection and inadequate storage conditions [14, 15].

**Table 1.** Mean mycotoxins level in recently harvested grains in Idah region

Mycotoxin type		Mycotoxin level (µg kg^−1^) in grains			
		Maize	Rice	Sorghum	Millet
Aflatoxins	B1	1±0.2^b^	0±0.0^a^	3±0.3^d^	2±0.2^b^
	B2	0±0.0^a^	0±0.0^a^	2±0.2^b^	0±0.0^a^
	G1 G2	0±0.0^a^ 0±0.0^a^	0±0.0^a^ 0±0.0^a^	0±0.0^a^ 0±0.0^a^	0±0.0^a^ 1±0.2^b^
Trichothecenes	Deoxynivalenol	520±0^e^	0±0.0^a^	400±0.7^g^	0±0.0^a^
	Zearalenone	250±0.4^f^	0±0.0^a^	250±0.4^f^	0±0.0^a^
Ochratoxins	Ochratoxin A	3±0.3^d^	0±0.0^a^	0±0.0^a^	0±0.0^a^
	Fumonisin	0±0.0^a^	0±0.0^a^	0±0.0^a^	0±0.0^a^
Patulin		0±0.0^a^	0±0.0^a^	0±0.0^a^	0±0.0^a^

Data are presented as means±SD. Means with different superscript within the same column and row are significantly different at *p*<.05.

Among the aflatoxins, aflatoxin B1 exhibited an increase in levels compared to recently harvested grains, with a mean level ranging from 3±0.3 µg kg^−1^ in maize, rice, sorghum, and millet. Aflatoxin B2 also showed higher levels in the stored grains, ranging from 2±0.2 µg kg^−1^ to 3±0.3 µg kg^−1^. Aflatoxin G1 and aflatoxin G2 were detected only in maize, with mean levels of 3±0.3 µg kg^−1^ and 3±0.3 µg kg^−1^, respectively. Regarding trichothecenes, deoxynivalenol levels increased in all grains during storage, with the highest mean level observed in maize at 700±1.2 µg kg^−1^. Zearalenone levels also exhibited an increase in maize, sorghum, and millet, with mean levels ranging from 200±0.4 µg kg^−1^ to 300±0.6 µg kg^−1^. Among the ochratoxins, ochratoxin A levels increased in maize and sorghum during storage, with mean levels of 4±0.3 µg kg^−1^ and 2±0.2 µg kg^−1^, respectively. Fumonisin and patulin were not detected in any of the grains.

Among the aflatoxins, aflatoxin B1 exhibited higher levels in maize and sorghum, with mean levels ranging from 3±0.3 µg kg^−1^ to 1±0.2 µg kg^−1^. Aflatoxin B2 showed variable levels across the grains, ranging from 2±0.2 µg kg^−1^ to 3±0.3 µg kg^−1^. Aflatoxin G1 and aflatoxin G2 were also detected, with varying levels across the grains. Trichothecenes, deoxynivalenol levels were observed in maize and sorghum, with a mean level of 400±0.7 µg kg^−1^. Zearalenone levels exhibited variability across the grains, ranging from 100±0.4 µg kg^−1^ to 270±0.5 µg kg^−1^.

Among the ochratoxins, ochratoxin A levels were detected in all grains, with mean levels ranging from 2±0.2 µg kg^−1^ to 3±0.3 µg kg^−1^. Fumonisin was detected only in rice, with a mean level of 2±0.2 µg kg^−1^. Patulin was not detected in any of the grains.

Among the aflatoxins, aflatoxin B1 exhibited higher levels in maize, rice, and sorghum, with mean levels ranging from 5±0.4 µg/ kg^−1^ to 4±0.3 µg kg^−1^. Aflatoxin B2 also showed elevated levels across the grains, ranging from 5±0.4 µg kg^−1^ to 4±0.3 µg kg^−1^. Aflatoxin G1 and aflatoxin G2 were detected in notable amounts as well. Trichothecenes, deoxynivalenol levels were observed in maize, sorghum, and millet, with a mean level of 700±1.2 µg kg^−1^. Zearalenone levels exhibited variability across the grains, ranging from 370±0.6 µg kg^−1^ to 200±0.4 µg kg^−1^. For the ochratoxins, ochratoxin A levels were detected in all grains, with mean levels ranging from 4±0.3 µg kg^−1^ to 2±0.2 µg kg^−1^. Fumonisin was not detected in any of the grains. Similarly, no traces of patulin were found.

Among the aflatoxins, aflatoxin B1 exhibited moderate levels in maize and sorghum, with mean levels of 2±0.2 µg kg^−1^. Aflatoxin B2 levels were also detected in maize, rice, and sorghum, with mean levels of 2±0.2 µg kg^−1^. Aflatoxin G1 and aflatoxin G2 were found in varying amounts across the grains. In terms of trichothecenes, deoxynivalenol levels were observed in maize and sorghum, with a mean level of 300±0.6 µg kg^−1^. Zearalenone levels showed variability, ranging from 200±0.4 µg kg^−1^ to 300±0.6 µg kg^−1^. Among the ochratoxins, ochratoxin A levels were detected in all grains, with mean levels ranging from 2±0.2 µg kg^−1^ to 3±0.3 µg kg^−1^. Fumonisin levels were also present in the grains, with mean levels ranging from 2±0.2 µg kg^−1^ to 3±0.3 µg kg^−1^. No traces of patulin were found.

Aflatoxin B1 levels were found in maize, rice, and sorghum, with mean levels ranging from 4±0.3 µg kg^−1^ to 5±0.3 µg kg^−1^. Aflatoxin B2 was detected in all grains, with mean levels ranging from 3±0.3 µg kg^−1^ to 4±0.3 µg kg^−1^. Aflatoxin G1 levels ranged from 3±0.3 µg kg^−1^ to 5±0.3 µg kg^−1^, while aflatoxin G2 levels were detected in sorghum and millet, with mean levels of 3±0.3 µg kg^−1^. Trichothecene mycotoxins were present in the stored grains, with deoxynivalenol levels ranging from 400±0.7 µg kg^−1^ to 450±0.7 µg kg^−1^. Zearalenone levels varied between 250±0.4 µg kg^−1^ and 400±0.7 µg kg^−1^. Among the ochratoxins, ochratoxin A levels were detected in sorghum and millet, with mean levels of 3±0.3 µg kg^−1^. Fumonisin levels were observed in sorghum and millet, with mean levels ranging from 3±0.3 µg kg^−1^ to 4±0.3 µg kg^−1^. No traces of patulin were found in the stored grains.

## Discussion

These findings underscore the significance of conducting regular mycotoxin analyses in grains sold in the Idah region. The observed contamination levels emphasize the need for effective measures to reduce mycotoxin levels and ensure the safety of grains consumed by the population.

Mycotoxin contamination in grains is a well-documented concern worldwide, as mycotoxins can have detrimental effects on human health and agricultural productivity. Studies have reported varying levels of mycotoxin contamination across different grains, which align with the data presented in Fig. 1. For instance, maize (corn) is known to be susceptible to mycotoxin contamination, particularly by aflatoxins, fumonisins, and deoxynivalenol (DON) [16]. Aflatoxins, produced primarily by *Aspergillus* fungi, are commonly found in maize and can pose significant health risks [17]. Additionally, fumonisins and DON, produced by *Fusarium* species, can also contaminate maize and have negative health effects [14]. Rice is generally considered less prone to mycotoxin contamination compared to maize. However, it can still be affected by mycotoxins such as aflatoxins, ochratoxin A, and zearalenone [18]. The reported contamination levels for rice in the given data (8%) align with the understanding that rice tends to have lower mycotoxin levels compared to other grains. Sorghum and millet, which are widely consumed in many regions, are susceptible to mycotoxin contamination as well. Sorghum can be contaminated with mycotoxins such as aflatoxins, fumonisins, and trichothecenes [19]. Millet, on the other hand, is known to be prone to contamination by aflatoxins and fumonisins [20].

It is important to note that mycotoxin contamination levels can vary depending on several factors, including geographical location, climatic conditions, agricultural practices, and storage conditions [16]. Therefore, it is crucial to conduct regular monitoring and analysis of mycotoxin levels in grains to ensure food safety.

Aflatoxins are highly toxic and can pose severe health risks, including hepatotoxicity and carcinogenicity [21]. The presence of these aflatoxins highlights the need for strict monitoring and control measures to ensure food safety and protect consumer health. The presence of other mycotoxins such as deoxynivalenol, ochratoxin A, and zearalenone also warrants attention. These mycotoxins can have adverse effects on human and animal health, including immune system disruption, nephrotoxicity, and estrogenic effects [14, 22].

It is essential to implement effective pre- and post-harvest management strategies, including good agricultural practices and proper storage conditions, to minimize mycotoxin contamination in grains. Additionally, regular monitoring programmes and adherence to regulatory standards are crucial for ensuring the safety of grains consumed in the Idah region.

The higher contamination rate during storage highlights the importance of proper post-harvest handling and storage practices to mitigate mycotoxin development. Factors such as temperature, moisture content, and storage duration can contribute to the growth of moulds and subsequent mycotoxin production [16]. Inadequate storage conditions and poor moisture control can promote fungal growth and mycotoxin accumulation in stored grains [23].

To minimize mycotoxin contamination during storage, it is essential to implement appropriate storage techniques, such as proper drying, cleaning, and the use of appropriate storage containers [16]. Effective measures like maintaining optimal moisture levels, ensuring proper ventilation, and periodic inspection of stored grains can help prevent or minimize mycotoxin formation.

During the harvest phase, contamination can occur due to pre-harvest factors such as fungal infection, weather conditions, and agricultural practices [23]. Timely and proper harvesting techniques, including avoiding harvesting when the crop is excessively wet or in adverse weather conditions, can help reduce pre-harvest mycotoxin contamination.

These results emphasize the need for comprehensive management strategies that encompass both pre-harvest and post-harvest measures to ensure grain quality and minimize mycotoxin contamination. It is crucial to educate farmers and stakeholders about best agricultural practices, as well as the importance of proper storage facilities and practices, to mitigate mycotoxin risks and safeguard food safety.

These results variations in mycotoxin contamination across different locations within the Idah region are due to factors such as geographical location, climate, agricultural practices, and storage conditions that can contribute to these variations [14, 19]. It is important to consider these factors when implementing strategies to mitigate mycotoxin risks and ensure food safety.

The implications of these results highlight the need for location-specific monitoring and intervention strategies to address mycotoxin contamination effectively. Tailored approaches based on the specific mycotoxins prevalent in each location can help in developing targeted control measures and interventions. This may include implementing good agricultural practices, improving storage facilities, and promoting proper drying and handling techniques [14]. Regular monitoring of mycotoxin levels, coupled with proper awareness and education among farmers and stakeholders, is essential to mitigate mycotoxin risks and ensure the production of safe and high-quality grains in the Idah region.

The absence or low levels of certain mycotoxins, such as aflatoxin B2, aflatoxin G1, aflatoxin G2, fumonisin, and patulin, in the analysed grains may be attributed to factors such as the specific geographic region, prevailing climatic conditions, and agricultural practices [14].

The results emphasize the need for continuous monitoring and implementation of good agricultural and storage practices to mitigate mycotoxin contamination in grains. These practices include proper drying, cleaning, and storage conditions to prevent fungal growth and mycotoxin production [16, 23].

It is important to note that the mycotoxin levels reported in Table 1 are mean values and do not exceed the regulatory limits set by regulatory authorities. However, it is crucial to remain vigilant and continuously monitor mycotoxin levels in grains to ensure food safety and adherence to regulatory guidelines (FAO) [24]. Some countries like Brazil has recently focused more attention to legislation on mycotoxin contamination of grains to check occurrences above maximum limits and co-occurrence [25]. The results from Table 2 indicate a significant increase in mycotoxin levels during grain storage, which aligns with previous studies. These findings are consistent with research that has reported higher mycotoxin contamination in stored grains due to factors such as moisture content, temperature, and fungal growth [16, 26]. The elevated levels of aflatoxins, deoxynivalenol, and zearalenone emphasize the importance of implementing proper storage conditions to minimize mycotoxin formation. Adequate drying, cleaning, and storage practices are crucial to prevent moisture accumulation and fungal growth, thereby reducing mycotoxin contamination [16, 27]. It is worth noting that the mycotoxin levels reported in Table 2 comply with regulatory limits established by regulatory authorities. However, continuous monitoring and adherence to good storage practices remain vital to ensure food safety and compliance with regulatory guidelines [28, 24].

**Table 2.** Mean mycotoxins level in stored grains in Idah

Mycotoxin type		Mycotoxin level (µg kg^**−1**^) in grains			
		Maize	Rice	Sorghum	Millet
Aflatoxins	B1	3±0.3^c^	2±0.2^b^	4±0.3^c^	3±0.3^c^
	B2	2±0.2^b^	3±0.3^c^	3±0.3^c^	2±0.2^b^
	G1	3±0.3^c^	0±0.0^a^	0±0.0^a^	0±0.0^a^
	G2	3±0.3^c^	0±0.0^a^	0±0.0^a^	1±0.2^b^
Trichothecenes	Deoxynivalenol	700±1.2^g^	250±0.4^f^	400±0.7^e^	300±0.6^f^
Ochratoxins	Zearalenone	300±0.6^f^	200±0.4^d^	350±0.6^e^	0±0.0^a^
	Ochratoxin A	4±0.3^c^	0±0.0^a^	2±0.2^b^	2±0.2^b^
	Fumonisin	0±0.0^a^	0±0.0^a^	0±0.0^a^	0±0.0^a^
Patulin		0±0.0^a^	0±0.0^a^	0±0.0^a^	0±0.0^a^

Data are presented as means±SD. Mean with different superscript within the same column and row are significantly different at *p*<.05.

The results from Table 3 highlight the presence of mycotoxins in recently harvested grains in the Ajaka region. The variability in mycotoxin levels among the grains emphasizes the importance of monitoring and implementing good agricultural and storage practices to minimize mycotoxin contamination [16, 26]. The presence of aflatoxins, deoxynivalenol, zearalenone, ochratoxin A, fumonisin, and patulin in the harvested grains raises concerns for food safety. These mycotoxins have been associated with adverse health effects in humans and animals, including carcinogenicity, hepatotoxicity, and immunosuppression [16, 27].

**Table 3.** Mean mycotoxins levels in recently harvested grains in Ajaka

Mycotoxin type		Mycotoxin level (µg kg^**−1**^) in grains			
		Maize	Rice	Sorghum	Millet
Aflatoxins	B1	3±0.3^d^	2±0.2^b^	1±0.2^b^	1±0.2^b^
	B2	2±0.2^b^	3±0.3^d^	2±0.2^b^	o±o.o^a^
	G1	3±0.3^d^	0±0.0^a^	3±0.3^d^	2±0.2^b^
	G2	3±0.3^d^	0±0.0^a^	2±0.2^b^	1±0.2^b^
Trichothecenes	Deoxynivalenol	400±0.7^e^	0±0.0^a^	400±0.7^e^	0±0.0^a^
Ochratoxins	Zearalenone	270±0.5^g^	200±0.4^g^	100±0.4^f^	150±0.4^f^
	Ochratoxin A	3±0.3^d^	2±0.2^b^	3±0.3^d^	3±0.3^d^
	Fumonisin	0±0.0^a^	2±0.2^b^	0±0.0^a^	0±0.0^a^
Patulin		0±0.0^a^	0±0.0^a^	0±0.0^a^	0±0.0^a^

Data are presented as means±SD. Means with different superscript within the same column and row are significantly different at *p*<.05.

To mitigate mycotoxin contamination, it is essential to implement preventive measures such as proper drying, storage, and regular monitoring of agricultural commodities. Additionally, promoting awareness among farmers and providing training on good agricultural practices can contribute to reducing mycotoxin levels in grains [26, 27].

Further studies are warranted to investigate the specific factors contributing to the observed mycotoxin levels in the Ajaka region, including agricultural practices, climatic conditions, and storage facilities. This information can inform the development of targeted interventions to minimize mycotoxin contamination and ensure food safety.

The results from Table 4 indicate the presence of mycotoxins in stored grains in the Ajaka region. The higher levels of aflatoxins, deoxynivalenol, zearalenone, and ochratoxin A in the stored grains raise concerns for food safety. These mycotoxins have been associated with various health risks, including hepatotoxicity, nephrotoxicity, and reproductive disorders [16, 27].

**Table 4.** Mean mycotoxins level in stored grains in Ajaka

Mycotoxin type		Mycotoxin level (µg kg^−1^) in grains			
		Maize	Rice	Sorghum	Millet
Aflatoxins	B1	5±0.4^c^	3±0.3^b^	5±0.4^c^	4±0.3^c^
	B2	4±0.3^c^	3±0.3^b^	5±0.4^c^	5±0.4^c^
	G1	4±0.3^c^	0±0.0^a^	4±0.3^c^	3±0.3^b^
Trichothecenes	G2 Deoxynivalenol	5±0.4^c^ 700±1.2^g^	0±0.0^a^ 250±0.4^f^	5±0.4^c^ 600±1.2^g^	4±0.3^c^ 300±0.6^e^
	Zearalenone	370±0.6^e^	200±0.4^f^	200±0.4^f^	350±0.6^e^
Ochratoxins	Ochratoxin A	4±0.3^c^	2±0.2^b^	4±0.3^c^	3±0.3^b^
	Fumonisin	0±0.0^a^	0±0.0^a^	0±0.0^a^	0±0.0^a^
Patulin		0±0.0^a^	0±0.0^a^	0±0.0^a^	0±0.0^a^

Data are presented as rneans±SD. Mean with different superscript within the same column and row are significantly different at *p*<.05.

Further investigations are needed to explore the underlying factors contributing to the observed mycotoxin levels in the stored grains of the Ajaka region. Factors such as storage conditions, duration, and handling practices may influence mycotoxin development and accumulation [16, 27]. Continuous monitoring, research, and implementation of effective control measures are vital to safeguard the quality and safety of stored grains, protecting both human health and the economy of the region.

The results from Table 5 indicate the presence of mycotoxins in recently harvested grains in the Ogbogbo region. Although the mycotoxin levels are not as high as in some other regions, their presence highlights the need for preventive measures to ensure food safety. Ongoing research and efforts are necessary to develop and promote effective strategies to mitigate mycotoxin contamination, safeguarding both human health and the agricultural economy of the Ogbogbo region.

**Table 5.** Mean mycotoxin levels in recently harvested grains in Ogbogbo

Mycotoxin type		Mycotoxin level (µg kg^−1^) in grains			
		Maize	Rice	Sorghum	Millet
Aflatoxins	B1	2±0.2^b^	0±0.0^a^	2±0.l^b^	0±0.0^a^
	B2	2±0.2^b^	2±0.2^b^	2±0.2^b^	0±0.0^a^
	G1 G2	3±0.2^b^ 1±0.2^b^	3±0.3^b^ 0±0.0^a^	2±0.2^b^ 2±0.2^b^	2±0.2^b^ 2±0.2^b^
Trichothecenes	Deoxynivalenol Zearalenone	300±0.6^d^ 200±0.4^a^	0±0.0^a^ 0±0.0^a^	300±0.6^d^ 300±0.6^d^	0±0.0^a^ 250±0.4^d^
Ochratoxins	Ochratoxin A	2±0.2^b^	0±0.0^a^	2±0.2^b^	3±0.3^b^
	Fumonisin	2±0.2^b^	0±0.0^a^	3±0.3^b^	3±0.3^b^
Patulin		0±0.0^a^	0±0.0^a^	0±0.0^a^	0±0.0^a^

Data are presented as means±SD. Means with different superscript within the same column and row are significantly different at *p*<.05.

The results from Table 6 indicate the presence of mycotoxins in stored grains in the Ogbogbo region, emphasizing the potential risk of mycotoxin contamination during storage. These findings highlight the importance of implementing proper post-harvest handling and storage practices to prevent or reduce mycotoxin development. To mitigate mycotoxin contamination in stored grains, several strategies can be employed. These include proper drying, cleaning, and maintaining optimal moisture levels during storage [6]. Additionally, the use of appropriate storage structures, such as hermetic bags or airtight containers, can help create a favourable environment that limits fungal growth and mycotoxin production [29]. Furthermore, regular monitoring of mycotoxin levels in stored grains is crucial to ensure food safety and prevent potential health risks. This can be achieved through routine sampling and analysis of grains for mycotoxin presence [30]. This can be facilitated by rapid methods like fluorescence and optical spectroscopy that are useful for specific mycotoxins that have distinct fluorescence or absorption properties, offering advantages in terms of simplicity and speed. The choice between these techniques depends on the mycotoxin of interest, the sample matrix, and the required sensitivity and specificity. The concept of aptamer-based biosensing is being introduced as an innovation for the detection of toxic contaminants from foods, water, human fluids and the environment [31].

**Table 6.** Mean mycotoxins level in stored grains in Ogbogbo

Mycotoxin type		Mycotoxin level (µg kg^−1^) in grains			
		Maize	Rice	Sorghum	Millet
Aflatoxins	B1	5±0.3^c^	4±0.3^b^	5±0.3^c^	4±0.3^b^
	B2	4±0.3^b^	3±0.3^b^	5±0.3^c^	3±0.3^b^
	G1 G2	3±0.3^b^ 0±0.0^a^	4±0.3^c^ 0±0.0^a^	4±0.3^c^ 3±0.3^b^	5±0.3^c^ 3±0.3^b^
Trichothecenes	Deoxynivalenol Zearalenone	400±0.7^e^ 300±0.6^d^	0±0.0^a^ 0±0.0^a^	450±0.7^e^ 400±0.7^e^	420±0.7e 250±0.4^d^
Ochratoxins	Ochratoxin A	0±0.0^a^	0±0.0^a^	3±0.3^b^	3±0.3^b^
	Fumonisin	0±0.0^a^	0±0.0^a^	3±0.3^b^	4±0.3^c^
Patulin		0±0.0^a^	0±0.0^a^	0±0.0^a^	0±0.0^a^

Data are presented as means±SD. Means with different superscript within the same column and row are significantly different at *p*<.05.

Efforts should also be focused on raising awareness among farmers, traders, and consumers about the importance of proper storage practices and the potential health hazards associated with mycotoxin-contaminated grains. Key global actions for mycotoxin management in wheat and other small grains have been suggested [32].

The research findings provide valuable insights into the levels of mycotoxins in stored and recently harvested grains in different regions. The findings shed light on the extent of mycotoxin contamination in these grains and emphasize the importance of addressing this food safety issue. However, there are still knowledge gaps and areas that require further investigation.

One of the key aspects highlighted by this research is the presence of mycotoxins, such as aflatoxins, trichothecenes, zearalenone, ochratoxins, fumonisin, and patulin, in stored and recently harvested grains. These mycotoxins pose a significant risk to human and animal health, as they can have detrimental effects, including carcinogenic, teratogenic, immunosuppressive, and neurotoxic effects [33]. The detection of these mycotoxins in the studied grains raises concerns about the safety of the food supply and highlights the need for effective control measures. Furthermore, the variations in mycotoxin levels across different grains and regions indicate the importance of considering specific crop types and geographical locations when assessing mycotoxin contamination. Factors such as agricultural practices, climatic conditions, storage conditions, and fungal contamination levels can influence mycotoxin production and accumulation [34]. Therefore, a comprehensive understanding of the local context is crucial for implementing targeted strategies to mitigate mycotoxin contamination. Five keys to prevention and control of mycotoxins in grains have been proposed [35].

Despite the valuable insights provided by these studies, there are still knowledge gaps that need to be addressed. For instance, further research is needed to explore the specific fungal species responsible for mycotoxin production in the studied grains. Identifying the predominant fungal strains and understanding their ecological factors can contribute to developing effective control measures [36]. Additionally, investigating the effects of post-harvest and storage practices on mycotoxin contamination is essential. Good agricultural and post-harvest practices, including crop rotation, pest control, and regular monitoring, can contribute to minimizing mycotoxin levels [37, 38].

This includes evaluating the impact of drying methods, storage conditions, and handling practices on mycotoxin development. Developing and promoting best practices in these areas can significantly contribute to reducing mycotoxin levels in grains [29]. Moreover, there is a need for comprehensive risk assessment studies that consider the dietary exposure to mycotoxins through different food commodities. Understanding the cumulative exposure and potential health risks associated with the consumption of mycotoxin-contaminated grains can inform regulatory measures and public health interventions [39].

## Conclusion

In conclusion, the study provides important insights into mycotoxin contamination in stored and recently harvested grains. The findings underscore the need for effective control measures and highlight the significance of considering specific crop types and geographical locations in addressing mycotoxin contamination. Addressing the knowledge gaps through further research and risk assessment studies will contribute to enhancing food safety and safeguarding human and animal health.
